# Vitamin D Status, Muscle Strength and Physical Performance Decline in Very Old Adults: A Prospective Study

**DOI:** 10.3390/nu9040379

**Published:** 2017-04-13

**Authors:** Antoneta Granic, Tom R. Hill, Karen Davies, Carol Jagger, Ashley Adamson, Mario Siervo, Thomas B. L. Kirkwood, John C. Mathers, Avan A. Sayer

**Affiliations:** 1 Institute of Neuroscience, The Medical School, Newcastle University, Newcastle upon Tyne NE2 4HH, UK; antoneta.granic@newcastle.ac.uk (A.G.); karen.davies@newcastle.ac.uk (K.D.); 2 NIHR Newcastle Biomedical Research Centre, Newcastle University and Newcastle upon Tyne NHS Foundation Trust, Campus for Ageing and Vitality, Newcastle upon Tyne NE4 5PL, UK; 3 Newcastle University Institute for Ageing, Newcastle upon Tyne NE4 5PL, UK; carol.jagger@newcastle.ac.uk (C.J.); ashley.adamson@newcastle.ac.uk (A.A.); mario.siervo@newcastle.ac.uk (M.S.); tom.kirkwood@newcastle.ac.uk (T.B.L.K.); john.mathers@newcastle.ac.uk (J.C.M.); 4 Human Nutrition Research Centre, Newcastle University, Campus for Ageing and Vitality, Newcastle upon Tyne NE4 5PL, UK; tom.hill@newcastle.ac.uk; 5 School of Agriculture, Food and Rural Development, Kings Road, Newcastle University, Newcastle upon Tyne NE1 7RU, UK; 6 Institute for Health and Society, Newcastle University, Baddiley-Clark Building, Newcastle upon Tyne NE2 4AX, UK; 7 Institute of Cellular Medicine, Newcastle University, William Leech Building, Newcastle upon Tyne NE2 4HH, UK; 8 Institute for Cell and Molecular Biosciences, Newcastle University, Framlington Place, Newcastle upon Tyne NE2 4HH, UK

**Keywords:** 25(OH)D, muscle strength, physical performance, grip strength, Timed Up-and-Go Test, very old adults

## Abstract

Mixed reports exist about the role of 25-hydroxyvitamin D (25(OH)D) in muscle ageing and there are few prospective studies involving the very old (aged ≥ 85) who are at highest risk of low 25(OH)D, loss of muscle mass and strength, and physical performance decline. In the Newcastle 85+ Study (*n* = 845), we aimed to determine the association between 25(OH)D season-specific quartiles (hereafter SQ1–SQ4), grip strength (GS) and physical performance decline (Timed Up-and-Go Test, TUG) over 5 years using mixed models. In the time-only models with linear and quadratic slopes, SQ1 and SQ4 of 25(OH)D were associated with weaker GS initially in men (SQ1: β (SE) = −2.56 (0.96); SQ4: −2.16 (1.06)) and women (SQ1: −1.10 (0.52); SQ4: −1.28 (0.50)) (all *p* ≤ 0.04). In the fully adjusted models, only men in SQ1 had a significant annual decline in GS of 1.41 kg which accelerated over time (−0.40 (0.1)), (both *p* ≤ 0.003) compared with those in combined middle quartiles. Only women in SQ1 and SQ4 of 25(OH)D had worse TUG times initially, but the rate of TUG decline was not affected. Low baseline 25(OH)D may contribute to muscle strength decline in the very old and particularly in men.

## 1. Introduction

A number of recent epidemiological studies have indicated a role of serum 25-hydroxyvitamin D (25(OH)D) in the aetiology of health outcomes of older adults beyond skeletal health [1], including cognitive impairment [2,3], cancers, cardiovascular diseases [4,5], mortality [6], muscle weakness, gait disturbances and falls [7,8]. Identifying factors such as serum 25(OH)D [9] which may help to maintain or improve muscle strength, function, and physical performance into an advanced age in order to preserve independence, is potentially of considerable public health importance. 

Several lines of evidence have been suggested to support the involvement of 25(OH)D in skeletal muscle strength and function [7,8,9]. Firstly, clinical signs of severe 25(OH)D deficiency (<25 nmol/L) [10] have been linked to myopathy, muscle pain and impaired gait, with amelioration by vitamin D supplementation [9]. Secondly, several studies have localised vitamin D receptor (VDR) in human muscle cell lines, myoblasts [11], and adult skeletal muscle [12,13], although opposing views have been published [14]. Thirdly, functional in vitro studies, have provided insights into the direct biological role of the active form of 25(OH)D, 1,25(OH2)D in regulation of genes and signalling pathways affecting calcium homeostasis, proliferation and differentiation of muscle cells [9], and positive correlation between 25(OH)D_3_ and expression of 24 muscle genes at the mRNA level [13]. Fourthly, despite conflicting findings across individual intervention studies, results of the latest meta-analyses of randomized controlled trials (RCT) of vitamin D supplementation have showed a small but significant improvement in muscle strength and function in older adults who had 25(OH)D concentrations below 30 nmol/L [15] or 50 nmol/L [16], and a reduced risk of falls in those with 25(OH)D < 25 nmol/L at baseline after vitamin D and calcium co-administration [17]. Supplementation with calcifediol (20 µg over 6 months) improved appendicular lean mass, physical performance (Short Physical Performance Battery), 4-m gait speed, and reduced mean number of falls in post-menopausal women (aged 68 years) who were diagnosed with osteoporosis or had 25(OH)D concentration <75 nmol/L [18]. Lastly, results from observational studies [3,19], although inconsistent, have suggested that a 25(OH)D concentration of <50 nmol/L exerts a negative effect on various measures of muscle strength and function and physical performance in older adults aged ≥60.

However, there remains a debate. The latest report from the Scientific Advisory Committee on Nutrition (SACN, 2016) defined the threshold of 25 nmol/L of 25(OH)D as the “population protective” level for musculoskeletal health in the UK population, including older adults [20]. The US Institute of Medicine (IOM, 2011) did not support 25(OH)D concentrations >50 nmol/L (i.e., above deficiency threshold) as beneficial for non-skeletal health outcomes [21], recommending that further research was needed. In addition, there is emerging evidence of a non-linear (U- or J-shaped) relationship with risks at both low and high 25(OH)D for some outcomes [4,6,21]. Indeed, we have recently observed a U-shaped association between low and high 25(OH)D concentration and cognitive impairment, poor attention [22] and mortality [23] in very old participants in the Newcastle 85+ Study.

A survey of prospective studies that have examined the role of 25(OH)D in muscle strength and physical performance in older adults (e.g., [24,25,26,27,28,29]) showed that the studies differed with respect to participants’ characteristics, baseline 25(OH)D concentration, measures used to assess muscle strength and function, and baseline levels of these measures. Only a few have included the very old (aged ≥ 85) [24,26,27,29], despite this being the age group at greatest risk of muscle mass and strength loss [30,31], functional decline [32], and, perhaps, low 25(OH)D status [33].

Therefore, the aim of this study was to investigate the association between 25(OH)D concentration and muscle strength (grip strength, GS) and physical performance (Timed Up-and-Go Test, TUG) in very old adults over 5 years and to test the hypothesis that these may be non-linear relationships. 

## 2. Materials and Methods

### 2.1. Participants

Participants were members of the Newcastle 85+ Study, a longitudinal study of health trajectories and outcomes of a cohort born in 1921 and recruited through general practices (GP) in Newcastle and North Tyneside, UK. The study protocol, approvals, cohort characteristics and retention have been described previously [34,35,36]. Both multidimensional health assessments and GP medical records data were available for 845 participants at baseline (2006/07). Fasting blood samples for biomarkers analysis, including 25(OH)D were collected between June 2006 and September 2007 for 719 to 778 individuals (depending on the assay), and delivered within 1 h to the clinical biochemistry laboratory, Royal Victoria Infirmary, Newcastle, UK for processing [37]. Participants were followed up at 1.5 (wave 2), 3 (wave 3) and 5 years (wave 4). Of 845 participants, 754 (89.2%) had both 25(OH)D and GS, and 717 (84.9%) had both 25(OH)D and TUG data at baseline (wave 1). The study was approved by the Newcastle & North Tyneside Local Research Ethics Committee 1 [34], and conducted in accordance with the Declaration of Helsinki. Details of the study protocols and questionnaires can be found at http://research.ncl.ac.uk/85plus/. All participants provided their signed informed consent prior to study commencement or the consent was obtained from their consultee (usually a relative) if they lacked the capacity to consent.

### 2.2. Serum 25(OH)D

Total serum 25(OH)D concentration was measured by DiaSorin Radioimmune Assay (RIA) kit using 25OHD-specific antibodies and 125I-labelled 25(OH)D as a tracer (see Supplementary Materials for details). To account for seasonal variations in UVB exposure and vitamin D skin production [38], 25(OH)D was categorised into season-specific quartiles (hereafter SQ1–SQ4; see Supplementary Materials for cut-offs). Specifically, for the lowest season-specific 25(OH)D quartile (SQ1), 25(OH)D concentration ranged from 5–28 nmol/L in summer (June–August), 8–30 nmol/L in autumn (September–November), 6–22 nmol/L in winter (December–February), 5–17 nmol/L in spring (March–May). For the highest season-specific 25(OH)D quartile (SQ4), 25(OH)D concentration ranged from ≥69 nmol/L in summer, ≥62 nmol/L in autumn, ≥60 nmol/L in winter, and ≥47 nmol/L in spring [22,23]. Medium quartiles (SQ2 and SQ3) were combined and served as a referent. Pre-defined 25(OH)D cut-offs [5,10] were used in the sensitivity analysis to define severely deficient (<25 nmol/L) and sufficient (≥75 nmol/L) [10] group (combined middle categories served as a referent). The mean time between blood sampling and GS and TUG assessment was about 1 week (8.5 days).

### 2.3. Grip Strength

GS [39] was measured using a hand-held dynamometer (Takei A5401 digital 0–100 kg × 0.1 kd LCD). In a standing position and with the elbows at approximately 180° angle, participants were instructed to squeeze the dynamometer as hard as possible alternating between the hands. Two measurements (in kg) for each hand were obtained and the mean of four measurements for each participant (M, SD) was calculated [40] and used in the analysis.

### 2.4. Timed Up-and-Go Test

Physical performance was assessed by the TUG test [41]. The time needed to get up from a chair (seat height 46 cm from the floor), walk in straight line for 3 m to and back from a marker placed on the floor, and sit back on the chair was recorded in seconds (s) with a stopwatch. Each participant performed the test only once and the use of walking aids (e.g., cane, walking frame, and wheeled walker) was documented at each wave.

### 2.5. Potential Confounders

We considered the following confounders previously established in this cohort [42] and commonly reported in the literature in association with muscle strength and physical performance in older adults [43,44,45,46,47]. Socio-demographic factor included sex (binary).

Anthropometric factors were: (1) height (continuous) calculated from sex-specific demi-span formula to the nearest cm [35]; (2) fat-free mass (FFM; continuous (kg)) estimated from inbuilt precision equation of the Tanita-305 body fat bioimpedance instrument, Tanita Corp., Tokyo, Japan) [31]; (3) BMI (ordered) calculated as kg weight/m^2^ height and categorized as <18.5 (underweight)/>18.5 < 25 (normal)/>25 < 30 (overweight)/30 (obese); and (4) waist-hip ratio (continuous). Health-related factors were: (1) self-rated health compared to others of the same age (ordinal) categorized as excellent or very good/good/fair or poor; (2) number of chronic diseases (continuous), from the following list: arthritis (e.g., generalized osteoarthritis, rheumatoid osteoarthritis, etc.), hypertension, cardiac disease (e.g., heart failure, angina, myocardial infarction, coronary angioplasty, etc.), respiratory disease (e.g., bronchiectasis, pulmonary fibrosis, asthma, etc.), cerebrovascular disease (e.g., stroke, transient ischaemic attack, etc.), diabetes (Type 1, Type 2, and unspecified), cancer (any cancer diagnosis in the past 5 years excluding non-melanoma skin cancer) [22,35]; (3) renal impairment (yes/no) diagnosis determined by the Chronic Kidney Disease Epidemiology Collaboration guidelines [35]; (4) cognitive impairment (yes/no) for scoring <15 points on Standardized Mini-Mental State Examination (SMMSE) [35,46]; (5) having difficulty performing GS test due to arthritis (arthritis in one hand, both, one or more joints) (yes/no); (6) use of walking aids during TUG test (included cane, walking frame, and wheeled walker) (yes/no); and (7) retention (completing the study or not over 5 years to account for loss to follow-up due to death and withdrawal) (yes/no) [42].

Lifestyle factors were: (1) physical activity (ordinal) categorized as low/moderate/high (score 0–1/score 2–6/score 7–18, respectively), established through a purpose-designed questionnaire and derived from the frequency and intensity of physical activity per week [47].

25(OH)D status-related variables were: (1) season of blood draw (categorical): June–August (summer)/September–November (autumn)/December–February (winter)/March–May (spring) [35]; (2) taking vitamin D-containing supplements categorized as: yes, at least one/no but taking others/not taking any vitamin supplements (“taking others” included non-prescribed multivitamins, multivitamins with minerals, and other combination of vitamins except for vitamin D); (3) taking prescribed vitamin D medication (yes/no; “yes” included prescription vitamin D, calcium with vitamin D, bisphosphonate with calcium and vitamin D and others) [22,23].

### 2.6. Effect Modifier

We observed that trajectories of GS [42] and TUG differed by sex and that the intake of vitamin D supplements and prescribed medication was an important determinant of 25(OH)D concentration [31], and modifier of cognitive status [22] and longevity [23] in this cohort. Thus we conducted separate analyses in participants not taking vitamin D supplements and medication (hereafter “restricted cohort”, *n* = 678, of which 97.05% (*n* = 658) had complete GS and 25(OH)D, and 86.23% (*n* = 605) TUG and 25(OH)D data at baseline).

### 2.7. Statistical Analysis

We used linear mixed models to examine the association between 25(OH)D sex-specific quartiles and initial level and rate of change in GS and TUG over 5 years in all participants, in men and women separately, and in the restricted cohort. GS data were normally distributed, and TUG measurements were log_10_ transformed to correct for positive skew (log_10_ s). Time was scaled in years (continuous), and both linear and non-linear (quadratic; acceleration or deceleration in the rate of change) effects of time on GS and TUG trajectories were tested. All growth curve models included a random intercept and linear slope. 

Model 1 contained a linear trend of time (Time) and season-specific 25(OH)D quartiles. Model 2 was additionally adjusted for quadratic time (Time^2^), and interaction terms (Time ×25(OH)D, and Time^2^ × 25(OH)D) to test for non-linear trends and the rate of change by 25(OH)D groups over 5 years, respectively. Model 3 was further adjusted for sex, anthropometry (height and FFM, centred to sex-specific mean), health-related factors (cognitive impairment, total number of chronic diseases, and self-rated health), physical activity, and sex × Time interaction to test for sex differences in the rate of change in GS and TUG. TUG Model 3 was additionally adjusted for the use of walking aids at baseline and follow-up. Negative (positive) β estimates represent weaker (stronger) GS compared with the referent group. Increasing β estimates of TUG (log_10_ transformed) indicate worse/slower performance. 

#### Sensitivity Analysis

We compared participants with a complete GS, TUG and assigned 25(OH)D group data at baseline with those lost to follow-up (withdrawal or death) 5 years later by Mann-Whitney U tests for ordered and non-normally distributed continuous data, and by χ^2^ tests for categorical data.

To keep linear mixed models parsimonious, we included a set of common and previously established predictors of GS [42,43,44,45] and TUG [44,45] in the saturated model (Model 3), and additionally adjusted for the following in sensitivity analyses: renal impairment, BMI, waist-hip ratio, having difficulty performing GS test due to arthritis in hands, and retention (completing the study or not over 5 years). All mixed models were repeated with pre-defined 25(OH)D categories (lowest 25(OH)D: <25 nmol/L; highest: ≥75 nmol/L; combined middle categories served as a referent), and Model 2 was additionally adjusted for the season of blood draw. All analyses were conducted using IBM SPSS (V2.1; IBM Corporation, Armonk, NY, USA), with α = 0.05 (two-tailed).

## 3. Results

Participants’ characteristics by season-specific quartiles of 25(OH)D have been described previously [22,23], and are summarised in Table S1. Briefly, those in the highest 25(OH)D group were more likely to be women, to take vitamin D medication, to have an increased risk of prevalent cognitive impairment [22] and 6-year mortality [23], compared with those in the middle 25(OH)D group. Untransformed GS and TUG measurements by season-specific 25(OH)D quartiles and pre-defined 25(OH)D categories at baseline and follow-up in all participants, in men and women separately are presented in Table 1, and Table S2, respectively.

### 3.1. Season-Specific 25(OH)D and GS Decline

**Figure 1:**
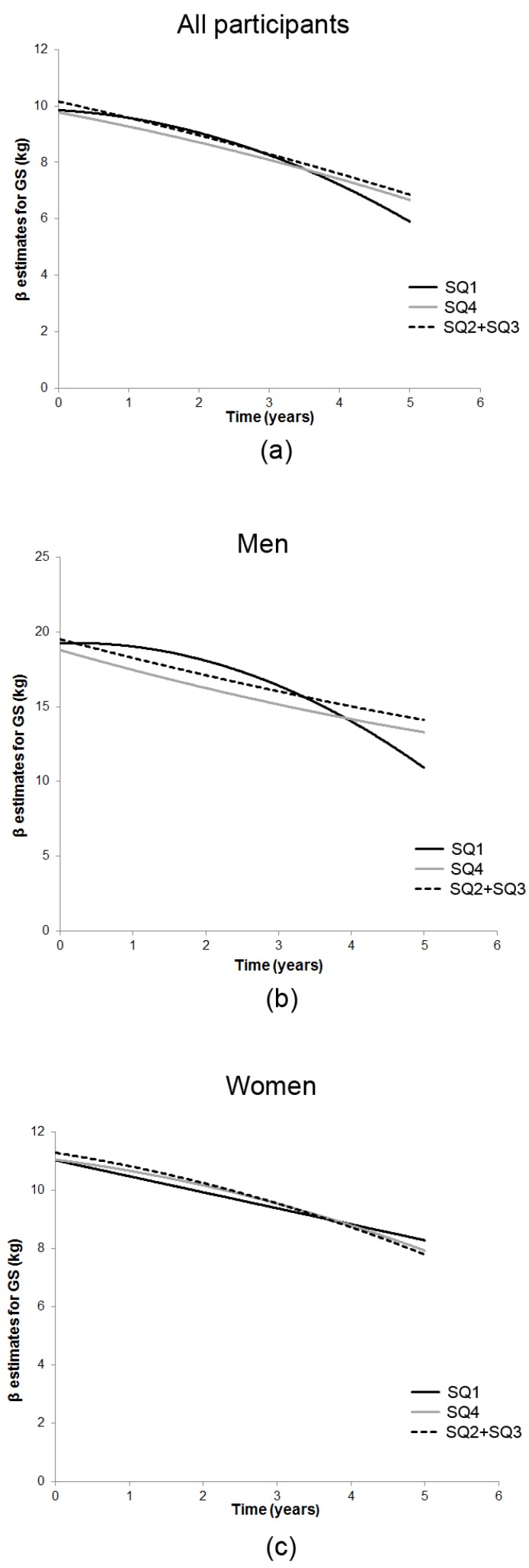
Estimated 5-year trajectories of grip strength (GS) by season-specific 25(OH)D quartiles in the Newcastle 85+ Study. In the model adjusted for key confounders (Model 3), participants in the lowest 25(OH)D quartile (SQ1, black solid line) had accelerated GS decline (**a**), whilst men in SQ1 (**b**) but not women (**c**) experienced a significant GS decline (1.41 kg/year) which accelerated over 5 years.

In the model with time (linear and quadratic), 25(OH)D and their interaction (Model 2, Table 2), we observed a U-shaped relationship between baseline GS and 25(OH)D groups in all participants, and for men and women separately. Specifically, both the lowest and highest season-specific 25(OH)D quartiles were associated with weaker GS in men (SQ1: β (SE) = −2.56 (0.96), *p* = 0.008; SQ4: −2.16 (1.06), *p* = 0.04) and women (SQ1: −1.10 (0.52), *p* = 0.04; SQ4: −1.28 (0.50), *p* = 0.01) compared with those in the middle quartiles. Only SQ1 was associated with a faster rate of GS decline in men, but not in women (Figure 1). Additionally, men in SQ1 experienced an accelerated GS decline of −0.44 kg annually over the 5-year follow-up (*p* < 0.001) (Model 2). In the restricted cohort, only SQ1 was associated with significantly weaker baseline GS and GS decline over time. After adjustments for key covariates (Model 3, Table 2, Figure 1), being in SQ1 was associated with GS decline of 1.41 kg (*p* = 0.003) per year which accelerated over time (−0.40 (0.1), *p* < 0.001) in men, but not in women. All participants in SQ1, as well as SQ1 participants who were also unsupplemented (restricted cohort), experienced accelerated GS decline over the follow-up period compared with participants belonging to combined middle quartiles (Figure 1).

### 3.2. Season-Specific 25(OH)D and Decline in TUG

**Figure 2:**
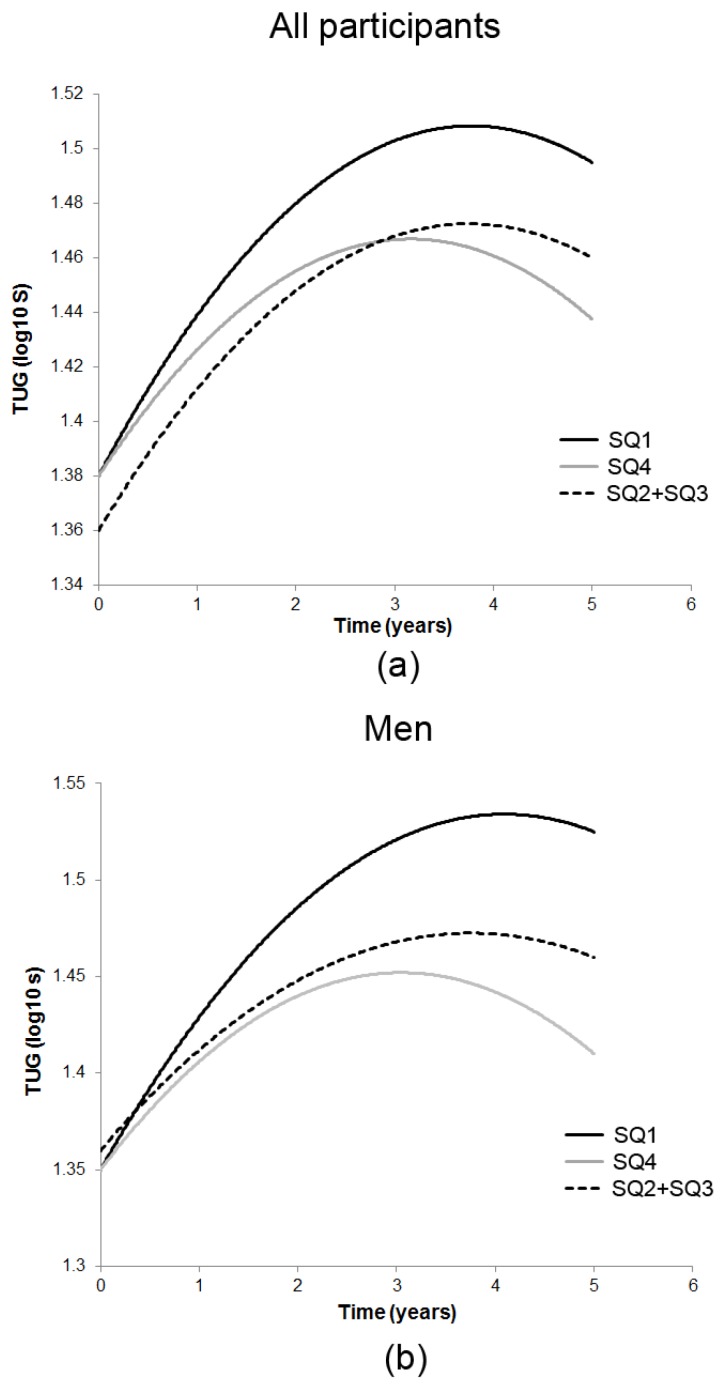

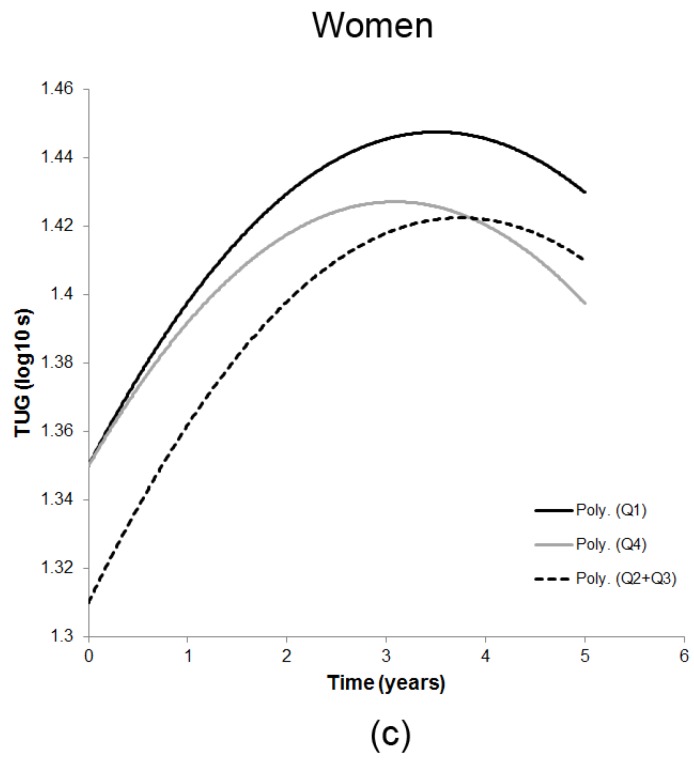
Estimated 5-year trajectories of Timed Up-and-Go test (TUG) by season-specific 25(OH)D quartiles in the Newcastle 85+ Study. In the model adjusted for key confounders (Model 3), no difference in the rate of decline in TUG over 5 years was observed in all participants (**a**), men (**b**) and women (**c**) across season-specific 25(OH)D quartiles (SQ1–SQ4). However, we observed a significant U-shaped association between baseline TUG and SQ1 (black solid line) and SQ4 (gray solid line) in all participants and in women compared with combined middle quartiles (SQ2 + SQ3) (dashed black line). Higher β estimates for TUG (log_10_ s) indicate worse (slower) performance (y-axes).

In the model with time (linear and quadratic), 25(OH)D and their interaction (Model 2, Table 3), a U-shaped association between baseline TUG and the lowest and highest season-specific 25(OH)D quartiles was observed in all participants and in women. The U-shaped relationship remained in women after adjustment for anthropometry, health-related variables and use of walking aids (SQ1: 0.04 (0.02), *p* = 0.04; SQ4: 0.04 (0.02), *p* = 0.03) (Model 3). However, the interaction terms between 25(OH)D quartiles and time were not significant indicating that the rate of decline in TUG did not differ by 25(OH)D group membership over 5 years in all participants, men and women (Figure 2). 

### 3.3. Results for Sensitivity Analysis

Compared to participants with complete data on both season-specific 25(OH)D and GS 5 years later (*n* = 286), those lost to follow-up (*n* = 468 (62.07%)) were more likely to be women (*p* = 0.04), to be cognitively impaired (*p* = 0.001) and depressed (*p* = 0.02), and less physically active (*p* = 0.02) at baseline. Similarly, compared to participants with complete data on 25(OH)D group and TUG 5 years later (*n* = 266), those lost to follow-up (*n* = 451 (62.9%)) were more likely to be cognitively impaired (*p* = 0.001), depressed (*p* = 0.02), and to be less physically active (*p* = 0.02) at baseline (data not shown).

#### 3.3.1. Pre-Defined 25OHD Categories and GS Decline

Overall, the association between GS and 25(OH)D obtained from the saturated models (Model 3, Supplemental Table S3) using pre-defined 25(OH)D categories (<25 nmol/L (lowest), 25–74 nmol/L (middle), and ≥75 nmol/L (highest)) were similar to those obtained with season-specific 25(OH)D quartiles. Briefly, the rate of decline in GS did not vary by 25(OH)D in all participants or women. Men in the lowest 25(OH)D category experienced GS decline of 1.23 kg per year (*p* = 0.01) which accelerated (−0.42 (0.10), *p* < 0.001) over time. Participants in the lowest category who were unsupplemented also experienced accelerated GS decline of −0.11 kg over the follow-up compared with participants belonging to the middle 25(OH)D category.

#### 3.3.2. Pre-Defined 25(OH)D Categories and Decline in TUG

Similarities and differences were observed in the results from Model 2 and 3 (Supplemental Table S4) for decline in TUG using pre-defined 25(OH)D categories compared with those using season-specific 25(OH)D quartiles (Table 3). In the models with time and 25(OH)D (Model 2), U-shaped relationships were confirmed in all participants and in women. However, in the fully adjusted model (Model 3) only the highest 25(OH)D category was associated with baseline TUG in women. Unlike the results from the main analysis (Model 3, Table 3), the rate of decline in TUG was affected by the membership of in the lowest (<25 nmol/L) 25(OH)D group in all participants and for those in the restricted cohort. In addition, a small U-shaped relationship between 25(OH)D categories and the rate of decline in TUG was observed in men. Compared with men in the middle 25(OH)D category (25–75 nmol/L), those in the lowest (<25 nmol/L) and the highest categories (≥75 nmol/L) had worse (slower) TUG performance with a slight deceleration over 5 years.

## 4. Discussion

The role of serum 25(OH)D in muscle strength and physical performance decline in older adults (aged ≥ 65) has been investigated intensively but has yielded inconclusive results [24,25,26,27,28,29,48,49]. To our knowledge, this is the first cohort study to test for non-linear relationships between 25(OH)D (defined by season-specific quartiles) and decline in GS and TUG in the very old (aged ≥ 85) living in the UK. We found a U-shaped association between 25(OH)D and GS at baseline in both men and women, and a significant association with GS decline in men in the lowest (SQ1) compared with combined middle 25(OH)D quartiles (SQ2 + SQ3) after adjustment for key covariates. Men in SQ1 experienced a loss of 1.41 kg/year and accelerated decline of −0.43 kg throughout the 5-year follow-up. Women (but not men) in the lowest and highest 25(OH)D season-specific quartile had worse (slower) overall TUG at baseline but not over time.

Prospective studies investigating the change in muscle performance with ageing in relation to serum 25(OH)D have been inconclusive [3]. Most have hypothesised a protective effect of higher 25(OH)D concentrations (≥50 or ≥75 nmol/L) for muscle health and functioning. Several have reported an increased risk of decline in participants with low vitamin D status (defined as either < 30 or < 50 nmol/L or lowest data-driven quartile) [24,25,26,27], whilst others have found no risk [49,50], or no association with the faster rate of decline in functioning measures over time [28,29]. Comparisons of our results with the findings from these studies are limited due to differences in serum 25(OH)D cut-offs, the specific muscle strength and physical performance tests used, length of follow-up, selection of confounders, and the small number of the very old included in the studies. We are aware of only one study of adults aged ≥80 from Belgium that found no association between 25(OH)D concentration and several measures of muscle performance in cross-sectional analyses, but interpretation of these findings may be complicated by the high prevalence of severe vitamin D deficiency (<25 nmol/L) in this cohort, especially in winter [48]. Because of pronounced seasonal variations in 25(OH)D in our study (51% had concentrations <30 nmol/L in spring, and 23% in autumn) [33], we used season-specific quartiles—a preferred method to adjust for the cyclical nature of 25(OH)D [38]. We also repeated the analysis for GS using pre-defined cut-offs [5,10], and did not find greater benefits for muscle strength in participants with 25(OH)D ≥75nmo/L.

In the UK, recently recommended 25(OH)D cut-offs both for overall and musculoskeletal health are much lower [20] than those proposed by the IOM [21] and the Endocrine Society guidelines [5,10] (25 vs. 50 vs. 75 nmol/L, respectively). The IOM also highlighted the emergence of evidence of a non-linear relationship between 25(OH)D and several extraskeletal outcomes [21], which we have reported for global cognition, attention [22], and mortality [23] in the very old. Greater benefits for cognition and longevity in this cohort were observed at concentrations between 40–60 nmol/L [22,23]. However, there remains a debate whether the U-shaped relationship between 25(OH)D and health outcomes could be biologically meaningful because the mechanisms for the apparent adverse effect of higher vitamin D status have been poorly understood or may reflect unmeasured confounding (e.g., hypovitaminosis D-related disease onset masked by supplementation) [51]. Future studies in this age group are needed to determine the thresholds for 25(OH)D concentration for different clinical and functional outcomes, and whether maintaining 25(OH)D between 40 to 60 nmol/L plays a role in healthy ageing in the very old [52]. 

Despite the differences in hypotheses, definitions of exposure (25(OH)D cut-offs), and outcome measures for muscle strength and function, there are certain parallels between the results found in our and other studies that included significant proportion the very old [26,27,28,29]. In a sub-group of 979 older adults (aged 65–88 years) from the Longitudinal Aging Study Amsterdam (LASA), those with 25(OH)D < 25 nmol/L had higher risk of decline in physical performance over 3 years, whilst those in the intermediate group (50–75 nmol/L) did not experience greater rates of decline compared with participants with 25(OH)D > 75 nmol/L [26]. Also, varying 25(OH)D thresholds across different health outcomes, gender, and age groups (55–85 years) have been found in LASA participants, which were lower in women and the oldest old (≥75 years) [52]. Using data from the Health, Aging, and Body Composition Study of over 2600 older adults aged 71–80, Houston et al. (2012) proposed thresholds and best performance concentrations of 25(OH)D for physical function and strength at 70–80 nmol/L and 55–70 nmol/L, respectively [28]. Although participants with 25(OH)D < 50 nmol/L had worse physical performance at baseline and at 2- and 4-years follow-up compared with those in sufficient group (≥75 nmol/L), no association was found for GS, and no association with a faster rate of decline in either measure. Taken together, the results suggest detrimental effects of low serum 25(OH)D (<25 nmol/L) and no change (decline) or favourable outcomes for muscle strength and physical performance at both intermediate (>50 nmol/L) and higher (>75 nmol/L) concentrations. In the very old (aged ≥ 85), we observed faster GS decline in SQ1 (the lowest value range: 17–30 nmol/L), especially in men, and no beneficial effect in SQ4 (the highest value range: ≥47 to >69 nmol/L). Also, men in both the severely deficient (<25 nmol/L) and sufficient (≥75 nmol/L) groups had worse (slower) performance in TUG over 5 years with a slight deceleration, possibly explained by the selective mortality of less healthy men. Women in both SQ1 and SQ4 and those with 25(OH)D ≥ 75nmol/L had worse initial TUG times, but no differences over time. Therefore, keeping 25(OH)D above the 25–30 nmol/L minimum may reduce muscle strength decline, whereas values >50 nmol/L may not confer additional benefits for muscle heath and musculoskeletal function in the very old. 

Lower baseline 25(OH)D was more relevant for muscle strength decline in men than in women after adjustment for a range of confounders, including physical activity, disease burden, renal impairment and retention (in sensitivity analysis; data not shown). Greater vitamin D supplementation explained the higher (mean) 25(OH)D concentration in women than in men (47.07 vs. 42.88 nmol/L, respectively), and no sex differences were observed in the restricted cohort. We have previously described sex-specific trajectories and baseline determinants of GS decline over 5 years in the very old [42]. Steeper slopes of GS decline in men compared with women could be explained partially by multi-morbidity [35,46] (a significant predictor of weaker GS in women), body composition [31] (fat mass was higher in women despite lower body weight), and survival. We have also reported shorter survival in women in both low and high 25(OH)D groups [23]. In addition, as in all studies of very old individuals, women’s longer life expectancy spent with more diseases and disabilities [46], and selective mortality in men (survival of healthier men), may have resulted in a biased sample, and a lack of power to detect associations in women.

The intake of vitamin D supplements and medication was an important determinant of 25(OH)D status in this cohort [22,23,33], especially in women, and was mainly related to diagnosis of osteoporosis [35]. However, similar acceleration in the rate of GS decline was observed in all participants and in those who were not supplemented with vitamin D, suggesting that supplementation did not attenuate the findings and that other sources of vitamin D (diet and sun exposure) may be more relevant for musculoskeletal health. Although recent meta-analyses of RCT have reported small improvements in muscle strength and function in deficient older adults (25(OH)D < 30 or 50 nmol/L) [15,16], larger scale studies [18] are needed to determine appropriate sources and thresholds, of 25(OH)D to maintain good musculoskeletal function in advanced adulthood.

### Strengths and Limitations

The results of our study should be interpreted with caution. The study is observational and does not imply causality between low 25(OH)D and worse muscle strength/physical performance. Older adults with poor physical function at baseline may have had lower 25(OH)D for reasons which were not included in the mixed models (e.g., frailty, sun exposure or polypharmacy). Therefore, the findings may be confounded by unmeasured or uncontrolled factors increasing the chance of Type I error. For example, we did not control for other 25(OH)D-related hormones (e.g., parathyroid hormone, PTH) and measures of bone health (e.g., bone mineral density), which have been implicated in the increased risk of sarcopenia (i.e., progressive loss of muscle mass and strength) [53], and higher GS and lean mass in older adults [54], respectively. On the other hand, adding more confounders to the fully adjusted model may have resulted in non-significant (bias) result and reduced power to detect significant associations, given the fact that each season-specific 25(OH)D quartile had, on average, 194 participants. Although we adjusted for fat-free mass in the analyses, the variable was estimated using the Tanita-305 bioimpedance instrument, and dual-energy X-ray absorptiometry (DXA) or magnetic resonance imaging would be a preferable method to reduce the risk of overestimation of lean and underestimation of fat mass [55].

There were several potential limitations related to the characterization of vitamin D status which may have increased the risk of mis-classification of exposure. Specifically, since 25(OH)D status prior to baseline was unknown, we could not adjust for long-standing vitamin D deficiency (which may have been corrected by supplementation prior to study commencement). Also, dosage and duration of vitamin D supplementation and potential interactions with other medication were unknown. Whilst we used a well-established method to account for the cyclic nature of 25(OH)D concentration across the year [38], a single measure may mis-classify status for individuals throughout the year. Because 25(OH)D status was established only at baseline (2006/07) for each participant, the significance of 25(OH)D fluctuation (from winter to summer months and over the follow-up) for muscle function could not be explored. Our choice of 25(OH)D assay (DiaSorin) has been reported to overestimate 25(OH)D deficiency (<30 nmol/L) [56] compared with some other methods (i.e., liquid chromatography tandem-mass spectrometry, LC-MS/MS), particularly in older women [57]. However, this is unlikely to have affected ranking of vitamin D status within sexes. Therefore, the nature and shape of the relationship between 25(OD)D and functional outcomes that we have observed are likely to be robust, albeit that their location on the 25(OH)D continuum may need to be confirmed by alternative 25(OH)D quantification (e.g., LC-MS/MS). The exact sun exposure (duration, use of sunscreen and protective clothing) in this cohort was unknown, and we used physical activity as a proxy. Whilst recognizing its limitations, there is a good evidence that greater physical activity is associated with higher vitamin D status [58]. In summary, all the above factors may have contributed to overestimation of low 25(OH)D in the very old, and, consequently, affected estimation of the precise 25(OH)D concentration ranges where there are association with GS and TUG. Carefully designed RCTs with similar population of the very old and longitudinal follow-up would be needed to test the 25(OH)D-muscle function hypothesis.

Further limitations of the study include its limited generalisability to the white population aged ≥85 living at similar latitudes (55° N). In studies of this kind, loss to follow-up due to high mortality among the very old, and the presence of more robust survivors in the sample, is unavoidable limitation. We observed that participants remaining in the study were healthier (less cognitive impairment, depression and fewer chronic diseases), but had similar 25(OH)D levels compared with participants lost to follow-up [23]. In addition, the relatively small β estimates for TUG may not represent clinically relevant changes in this function.

Our study also had a number of strengths including its prospective design using a single birth cohort (homogenous age); a broad representativeness of the general population in England and Wales; stratified analyses by sex and exposure (including estimates of vitamin D supplementation); use of season-specific 25(OH)D cut-off values to adjust for the cyclic nature of 25(OH)D [38], and adjustment for previously established determinants of muscle strength decline [42] in the multilevel analyses.

## 5. Conclusions

We have found that the lowest 25(OH)D season-specific quartile was associated with a faster rate of muscle strength (GS) decline in men (aged ≥ 85), and acceleration of the decline over 5 years in all participants as well as those not supplemented with vitamin D. The rate of decline in physical performance (TUG) did not differ across the vitamin D quartiles. Serum 25(OH)D may be an important predictor of multiple health outcomes, including musculoskeletal health in the very old. These results need to be corroborated in other prospective studies of this age group to aid definitive trials of 25(OH)D for musculoskeletal health in later life.

## Figures and Tables

**Table 1:** Grip strength and Timed Up-and-Go test measurements by season-specific 25(OH)D quartiles over 5 years ^†^.

Measure/Time of Assessment ^‡^	*n*	SQ1 25(OH)D	SQ2 + SQ3 25(OH)D	SQ4 25(OH)D
		Lowest	Middle	Highest
*Grip strength, kg (SD)*				
**All participants**				
Baseline	754	16.83 (6.80)	19.20 (8.13)	15.92 (7.29)
1.5-year follow-up	582	16.35 (7.73)	18.26 (7.86)	15.21 (7.63)
3-year follow-up	434	16.26 (7.16)	17.26 (7.46)	15.26 (6.92)
5-year follow-up	286	13.66 (6.15)	15.64 (7.38)	14.58 (6.83)
**Men**				
Baseline	301	23.12 (5.43)	25.44 (7.14)	23.67 (6.65)
1.5-year follow-up	224	23.43 (6.80)	24.39 (7.12)	23.01 (7.72)
3-year follow-up	163	22.79 (6.24)	23.05 (6.85)	22.91 (7.35)
5-year follow-up	104	17.41 (7.57)	22.01 (6.48)	22.32 (6.77)
**Women**				
Baseline	453	12.92 (4.10)	13.97 (4.33)	12.68 (4.61)
1.5-year follow-up	358	12.29 (4.72)	13.38 (4.13)	12.21 (5.05)
3-year follow-up	271	12.63 (4.63)	12.96 (4.34)	12.45 (4.08)
5-year follow-up	182	12.63 (4.63)	11.39 (4.21)	11.76 (4.21)
*Timed Up-and-Go Test, s (SD)*				
**All participants**				
Baseline	717	20.93 (17.39)	16.75 (13.32)	19.76 (13.72)
1.5-year follow-up	529	22.48 (14.53)	20.06 (15.53)	22.14 (14.51)
3-year follow-up	389	24.99 (25.67)	19.70 (14.08)	22.08 (20.26)
5-year follow-up	266	24.37 (16.44)	19.88 (10.55)	19.71 (11.56)
**Men**				
Baseline	287	18.99 (19.00)	15.06 (11.65)	15.76 (8.83)
1.5-year follow-up	210	22.23 (16.97)	18.47 (13.78)	16.57 (6.86)
3-year follow-up	149	22.50 (20.78)	16.91 (8.23)	22.58 (32.58)
5-year follow-up	94	19.71 (11.04)	17.92 (9.77)	15.83 (6.43)
**Women**				
Baseline	430	22.15 (18.78)	18.14 (14.43)	21.54 (15.10)
1.5-year follow-up	319	22.63 (12.95)	21.33 (16.74)	24.56 (16.24)
3-year follow-up	240	26.37 (28.13)	21.74 (16.87)	21.86 (11.71)
5-year follow-up	172	26.15 (17.91)	21.14 (10.89)	21.41 (12.89)

^†^ Season-specific quartiles of 25(OH)D (SQ1–SQ4) [24,25] (see Supplementary Materials for cut-offs). The middle SQ2 and SQ3 were combined to form three season-specific 25(OH)D groups: lowest (SQ1), middle (SQ2 + SQ3), and highest (SQ4). ^‡^ Untransformed data.

**Table 2:** β estimates of grip strength by season-specific 25(OH)D quartiles over 5 years.

Outcome	Effects/Variable	Model 1		Model 2		Model 3	
		β (SE) ^†^	*p*	β (SE) ^†^	*p*	β (SE) ^†^	*p*
*All participants*							
GS (kg)	Intercept	19.10 (0.38)	<0.001	19.14 (0.39)	<0.001	10.16 (0.75)	<0.001
	25(OH)D quartiles						
	Lowest (SQ1)	−2.24 (0.65)	0.001	−2.48 (0.69)	<0.001	−0.31 (0.45)	0.49
	Middle (ref) (SQ2 + SQ3)	0		0		0	
	Highest (SQ4)	−3.06 (0.65)	<0.001	−3.37 (0.69)	<0.001	−0.39 (0.45)	0.39
GS decline ^‡^	Time	−0.80 (0.04)	<0.001	−0.74 (0.14)	<0.001	−0.56 (0.14)	<0.001
	Time^2^			−0.02 (0.03)	0.43	−0.02 (0.03)	0.5
Rate of decline	Slope ^§^						
	25(OH)D ×Time						
	Lowest × Time			0.56 (0.26)	0.03	0.42 (0.26)	0.1
	Middle × Time (ref)			0		0	
	Highest × Time			0.17 (0.25)	0.51	0.09 (0.26)	0.73
	25(OH)D × Time^2^						
	Lowest × Time^2^			−0.13 (0.05)	0.02	−0.11 (0.05)	0.03
	Middle × Time^2^			0		0	
	Highest × Time^2^			−0.001 (0.51)	0.99	−0.01 (0.05)	0.85
*Men*							
GS (kg)	Intercept	25.48 (0.51)	<0.001	25.50 (0.51)	<0.001	19.5 (1.46)	<0.001
	25(OH)D quartiles						
	Lowest (SQ1)	−2.02 (0.93)	0.03	−2.56 (0.96)	0.008	−0.25 (0.89)	0.78
	Middle (ref) (SQ2 + SQ3)	0		0		0	
	Highest (SQ4)	−2.12 (1.03)	0.04	−2.16 (1.06)	0.04	−0.89 (0.96)	0.35
GS decline ^‡^	Time	−1.10 (0.08)	<0.001	−1.18 (0.24)	<0.001	−1.28 (0.23)	<0.001
	Time^2^			0.02 (0.05)	0.64	0.04 (0.05)	0.4
Rate of decline	Slope ^§^						
	25(OH)D × Time						
	Lowest × Time			1.71 (0.46)	<0.001	1.41 (0.47)	0.003
	Middle × Time (ref)			0		0	
	Highest × Time			0.02 (0.51)	0.03	−0.12 (0.52)	0.82
	25(OH)D × Time^2^						
	Lowest × Time^2^			−0.44 (0.09)	<0.001	−0.40 (0.1)	<0.001
	Middle × Time^2^			0		0	
	Highest × Time^2^			0.01 (0.11)	0.91	0.03 (0.1)	0.77
*Women*							
GS (kg)	Intercept	13.94 (0.29)	<0.001	13.88 (0.31)	<0.001	11.30 (0.75)	<0.001
	25(OH)D quartiles						
	Lowest (SQ1)	−1.08 (0.48)	0.03	−1.10 (0.52)	0.04	−0.26 (0.48)	0.59
	Middle (ref) (SQ2 + SQ3)	0		0		0	
	Highest (SQ4)	−1.14 (0.46)	0.01	−1.28 (0.50)	0.01	−0.24 (0.45)	0.60
GS decline ^‡^	Time	−0.59 (0.05)	<0.001	−0.33 (0.16)	0.05	−0.40 (0.17)	0.02
	Time^2^			−0.07 (0.03)	0.04	−0.06 (0.03)	0.07
Rate of decline	Slope ^§^						
	25(OH)D × Time						
	Lowest × Time			−0.17 (0.30)	0.56	−0.15 (0.30)	0.62
	Middle × Time			0		0	
	Highest × Time			0.05 (0.28)	0.85	0.08 (0.28)	0.79
	25(OH)D × Time^2^						
	Lowest × Time^2^			0.06 (0.06)	0.35	0.06 (0.06)	0.37
	Middle × Time^2^			0		0	
	Highest × Time^2^			0.01 (0.06)	0.83	−0.001 (0.06)	0.98
*Restricted cohort*							
GS (kg)	Intercept	19.6 (0.41)	<0.001	19.6 (0.43)	<0.001	10.23 (0.85)	<0.001
	25(OH)D quartiles						
	Lowest (SQ1)	−2.76 (0.68)	<0.001	−2.95 (0.72)	<0.001	−0.35 (0.48)	0.47
	Middle (ref) (SQ2 + SQ3)	0		0		0	
	Highest (SQ4)	−1.08 (0.88)	0.22	−1.25 (0.93)	0.18	−0.35 (0.59)	0.55
GS decline ^‡^	Time			−0.75 (0.15)	<0.001	−0.54 (0.15)	0.001
	Time^2^			−0.02 (0.03)	0.5	−0.02 (0.03)	0.52
Rate of decline	Slope ^§^						
	25(OH)D × Time						
	Lowest × Time			0.52 (0.27)	0.05	0.36 (0.27)	0.19
	Middle × Time (ref)			0		0	
	Highest × Time			0.07 (0.33)	0.84	0.04 (0.33)	0.91
	25(OH)D × Time^2^						
	Lowest × Time^2^			−0.12 (0.05)	0.02	−0.11 (0.05)	0.05
	Middle × Time^2^			0		0	
	Highest × Time^2^			0.003 (0.06)	0.96	0.002 (0.06)	0.97

^†^ β-coefficients (SE) are estimates of fixed effects with longitudinal GS data to evaluate population averages in GS. Fixed effects of covariates estimated initial level and trajectory differences in GS as a function of the covariate in the model. ^‡^ The main effect of time (Time and Time^2^) tested linear and non-linear (quadratic) change in GS over 5 years. ^§^ Interaction terms tested whether GS slopes varied by the covariate over 5 years. Model 1 includes a linear trend of time and season-specific 25(OH)D quartiles. Model 2 is additionally adjusted for quadratic trend of time and interaction terms (Time × 25(OH)D quartiles, Time^2^ × 25(OH)D quartiles). Model 3 is further adjusted for sex, anthropometry (height and FFM), health-related variables (cognitive impairment, disease count, self-rated health), physical activity, and interaction term (sex × Time) (except in men and women).

**Table 3:** β Estimates of Timed Up-and-Go test by season-specific 25(OH)D quartiles over 5 years.

Outcome	Effects/Variable	Model 1		Model 2		Model 3	
		β (SE) ^†^	*p*	β (SE) ^†^	*p*	β (SE) ^†^	*p*
*All participants*							
TUG (log_10_ s)	Intercept	1.17 (0.01)		1.16 (0.01)	<0.001	1.56 (0.03)	<0.001
	25(OH)D quartiles						
	Lowest (SQ1)	0.09 (0.02)	<0.001	0.08 (0.02)	<0.001	0.02 (0.02)	0.23
	Middle (ref) (SQ2 + SQ3)	0		0		0	
	Highest (SQ4)	0.06 (0.02)	0.005	0.07 (0.02)	0.003	0.02 (0.02)	0.17
TUG decline ^‡^	Time	0.03 (0.002)	<0.001	0.06 (0.006)	<0.001	0.06 (0.01)	<0.001
	Time^2^			−0.01 ((0.001)	<0.001	−0.01 (0.001)	<0.001
Rate of decline	Slopes ^§^						
	25(OH)D × Time						
	Lowest × Time			0.01 (0.01)	0.58	0.01 (0.01)	0.51
	Middle × Time (ref)			0		0	
	Highest × Time			−0.005 (0.01)	0.65	−0.01 (0.01)	0.63
	25(OH)D × Time^2^						
	Lowest × Time^2^			−0.0001 (0.002)	0.97	−0.001 (0.002)	0.62
	Middle × Time^2^			0		0	
	Highest × Time^2^			−0.001 (0.002)	0.71	−0.001 (0.002)	0.77
*Men*							
TUG (log_10_ s)	Intercept	1.13 (0.02)	<0.001	1.12 (0.02)	<0.001	1.57 (0.04)	<0.001
	25(OH)D quartiles						
	Lowest (SQ1)	0.09 (0.03)	0.002	0.09 (0.03)	0.006	−0.01 (0.03)	0.69
	Middle (ref) (SQ2 + SQ3)	0		0		0	
	Highest (SQ4)	0.03 (0.03)	0.78	0.03 (0.03)	0.42	−0.01 (0.03)	0.68
TUG decline ^‡^	Time	0.04 (0.003)	<0.001	0.06 (0.01)	<0.001	0.06 (0.01)	<0.001
	Time^2^			−0.01 (0.001)	0.001	−0.01 (0.001)	<0.001
Rate of decline	Slopes ^§^						
	25(OH)D × Time						
	Lowest × Time			0.02 (0.02)	0.22	−0.003 (0.004)	0.41
	Middle × Time (ref)			0		0	
	Highest × Time			0.001 (0.002)	0.96	−0.003 (0.004)	0.47
	25(OH)D × Time^2^						
	Lowest × Time^2^			−0.002 (0.004)	0.61	−0.01 (0.001)	0.41
	Middle × Time^2^			0		0	
	Highest × Time^2^			−0.002 (0.004)	0.57	−0.003 (0.004)	0.47
*Women*							
TUG (log_10_ s)	Intercept	1.21 (0.02)	<0.001	1.19 (0.02)	<0.001	1.51 (0.03)	<0.001
	25(OH)D quartiles						
	Lowest (SQ1)	0.07 (0.03)	0.007	0.07 (0.03)	0.01	0.04 (0.02)	0.04
	Middle (ref) (SQ2 + SQ3)	0		0		0	
	Highest (SQ4)	0.06 (0.03)	0.03	0.07 (0.03)	0.02	0.04 (0.02)	0.03
TUG decline ^‡^	Time	0.03 (0.003)	<0.001	0.06 (0.01)	<0.001	0.06 (0.01)	<0.001
	Time^2^			0.006 (0.002)	<0.001	−0.01 (0.002)	<0.001
Rate of decline	Slope ^§^						
	25(OH)D × Time						
	Lowest × Time			−0.003 (0.02)	0.86	−0.004 (0.02)	0.8
	Middle × Time			0		0	
	Highest × Time			−0.01 (0.01)	0.59	−0.01 (0.01)	0.45
	25(OH)D × Time^2^						
	Lowest × Time^2^			0.001 (0.003)	0.7	0.00001 (0.003)	0.99
	Middle × Time^2^			0		0	
	Highest × Time^2^			−0.0004 (0.003)	0.9	0.0001 (0.003)	0.97
*Restricted cohort*							
TUG (log_10_ s)	Intercept	1.16 (0.01)	<0.001	1.15 (0.01)	<0.001	1.55 (0.03)	<0.001
	25(OH)D quartile						
	Lowest (SQ1)	0.10 (0.02)	<0.001	0.10 (0.02)	<0.001	0.03 (0.02)	0.07
	Middle (ref) (SQ2 + SQ3)	0		0		0	
	Highest (SQ4)	-0.02 (0.02)	0.50	−0.02 (0.03)	0.56	−0.01 (0.02)	0.71
TUG decline ^‡^	Time	0.03 (0.002)	<0.001	0.06 (0.01)	<0.001	0.06 (0.01)	<0.001
	Time^2^			−0.01 (0.001)	<0.001	−0.01 (0.001)	<0.001
Rate of decline	Slope ^§^						
	25(OH)D × Time						
	Lowest × Time			0.006 (0.01)	0.61	0.01 (0.01)	0.52
	Middle × Time (ref)			0		0	
	Highest × Time			0.007 (0.01)	0.61	0.002 (0.01)	0.85
	25(OH)D × Time^2^						
	Lowest × Time^2^			0.0001 (0.002)	0.98	−0.001 (0.002)	0.61
	Middle × Time^2^			0		0	
	Highest × Time^2^			−0.003 (0.003)	0.26	−0.002 (0.003)	0.44

^†^ β-coefficients (SE) are estimates of fixed effects with longitudinal log_10_ transformed TUG data to evaluate population averages in TUG time. Fixed effects of covariates estimated initial level and trajectory differences in TUG as a function of the covariate in the model. ^‡^ The main effect of time (Time and Time^2^) tested linear and non-linear (quadratic) change in TUG over 5 years. ^§^ Interaction terms tested whether TUG slopes varied by the covariate over 5 years. Model 1 includes a linear trend of time and season-specific 25(OH)D quartiles. Model 2 is additionally adjusted for quadratic trend of time and interaction terms (Time × 25(OH)D quartiles, Time^2^ × 25(OH)D quartiles). Model 3 is further adjusted for sex, anthropometry (height and FFM), health-related variables (cognitive impairment, disease count, self-rated health), physical activity, use of walking aids during TUG testing (time-varying covariate) and interaction term (sex × Time).

## Supplementary Materials

The following are available online at http://www.mdpi.com/2072-6643/9/4/379/s1, Supplementary Methods: Serum 25(OH)D assay, Season-specific serum 25(OH)D quartiles cut-offs, Table S1: Characteristics of participants by season-specific 25(OH)D quartiles (summary), Table S2: Grip strength and Timed Up-and-Go test measurements by pre-defined 25(OH)D categories over 5 years, Table S3: β estimates of grip strength by pre-defined 25(OH)D categories over 5 years, Table S4: β estimates of Timed Up-and-Go test by pre-defined 25(OH)D categories over 5 years.
