# Supplementary material for: Vitamin D Status, Muscle Strength and Physical Performance Decline in Very Old Adults: A Prospective Study
Source: Nutrients. 2017 Apr 13;9(4):379. doi: 10.3390/nu9040379 (PMC5409718; doi:10.3390/nu9040379)
Supplement: Supplementary file 1 [file nutrients-09-00379-s001.docx]

**Supplementary Materials**

**1. Supplementary Methods**

*1.1. Serum 25(OH)D Assay*

Serum 25-hydroxyvitamin D (25(OH)D) concentration was determined by DiaSorin Radioimmune Assay (RIA) kit (DiaSorin Corporation, Stillwater, MN). The kit extracts 25-hydroxy-ergocalciferol (25(OH)D_2_), 25-hydroxy-cholecalciferol (25(OH)D_3_), and other hydroxylated vitamin D metabolites in two steps. After acetonitrile precipitation of two 50 μL blood serum aliquots of each sample (step 1), the extracted samples were assayed by RIA method with 25(OH)D-specific antibodies and ^125^I-labelled 25(OH)D (Diasorin Corporation) as a tracer (step 2). The sample, antibodies and tracer were co-incubated for 90 min at 20–25 °C, and phase-separated using the secondary antibody-precipitating complex for additional 20 min incubation at 20–25 °C. Prior to centrifugation, an NSB/Addition Buffer was added to prevent non-specific binding. The samples were measured using a Packard COBRA Quantum Gamma Counter (Packard Instrument Company, Downers Grove, IL, USA) and concentrations calculated with the accompanying software. The inter- and intra-assay coefficients of variation were between 8.4 to 12.6%.

*1.2. Season-Specific Serum 25(OH)D Quartiles Cut-Offs*

Season-specific serum 25(OH)D quartiles (hereafter SQ1 to SQ4) were created for each season of blood collection as follows.

*Summer* (June–August): SQ1 (5–28 nmol/L), SQ2 (29–45 nmol/L), SQ3 (46–68 nmol/L) and SQ4 (≥ 69 nmol/L).

*Autumn* (September–November): SQ1 (8–30 nmol/L), SQ2 (31–43 nmol/L), SQ3 (44–61 nmol/L), and SQ4 (≥ 62 nmol/L).

*Winter* (December–February): SQ1 (6–22 nmol/L), SQ2 (23–32 nmol/L), SQ3 (33–59 nmol/L), and SQ4 (≥ 60 nmol/L).

*Spring* (March–May): SQ1 (5–17 nmol/L), SQ2 (18–26 nmol/L), SQ3 (27–46 nmol/L), and SQ4 (≥ 47 nmol/L).

**Table S1.** Characteristics of participants by season-specific 25(OH)D quartiles (summary) *.

| **Lowest Season-Specific 25(OH)D Quartile** | **Middle Season-Specific 25(OH)D Quartiles** | **Highest Season-Specific 25(OH)D Quartile** |
| --- | --- | --- |
| SQ1 | SQ2+SQ3 | SQ4 |
| *n* = 191 | *n* = 392 | *n* = 192 |
| Most likely not to take any kind of supplements. | The least likely to be depressed. | Most likely to be women. |
| The least likely to drink alcohol. | The most physically active. | Most likely to take prescribed medication with |
| About 60% increased risk of prevalent global |  | vitamin D. |
| cognitive impairment and slower attention |  | Most likely to have osteoporosis. |
| reaction times compared with the middle 25(OH)D |  | The least likely to have cardiovascular diseases. |
| group. |  | 37% increased risk of 6-year mortality after |
|  |  | adjustment for sociodemographic and lifestyle |
|  |  | factors compared with the middle 25(OH)D group, |
|  |  | especially in women. |
|  |  | About 60% increased risk of prevalent global |
|  |  | cognitive impairment and slower attention |
|  |  | reaction times compared with the middle 25(OH)D |
|  |  | group. |

* Summarised from published results [22,23].

**Table S2.** Grip strength and Timed Up-and-Go test measurements by pre-defined 25(OH)D categories * over 5 years.

_________________________________________________________________________________________________________________________________________

**Measure / time of assessment** ***n*** **Lowest 25(OH)D** **Middle 25(OH)D** **Highest 25(OH)D**

_________________________________________________________________________________________________________________________________________

Pre-defined cut-offs* < 25 nmol/L 25–74 nmol/L ≥ 75 nmol/L

*n* (%) 191 (24.6) 469 (60.5) 115 (14.8)

*Grip strength, kg (SD)*

**All participants**

Baseline 754 16.40 (6.85) 18.89 (8.13) 15.73 (6.67)

1.5-year follow-up 582 15.85 (7.58) 18.18 (7.88) 14.40 (7.46)

3-year follow-up 434 15.27 (7.00) 17.45 (7.39) 14.50 (6.73)

5-year follow-up 286 12.58 (5.94) 15.79 (7.32) 14.43 (6.27)

**Men**

Baseline 301 22.93 (5.66) 25.22 (7.07) 23.75 (6.07)

1.5-year follow-up 224 23.42 (6.79) 24.32 (7.04) 22.09 (8.76)

3-year follow-up 163 21.98 (6.27) 23.28 (6.88) 22.72 (7.29)

5-year follow-up 104 17.34 (7.79) 21.83 (6.79) 21.68 (5.84)

**Women**

Baseline 453 12.73 (4.23) 13.75 (4.51) 12.92 (4.12)

1.5-year follow-up 358 12.07 (4.50) 13.38 (4.40) 11.96 (4.99)

3-year follow-up 271 11.91 (4.49) 13.26 (4.24) 12.09 (4.24)

5-year follow-up 182 10.74 (4.10) 11.68 (4.14) 12.01 (4.27)

*Timed Up-and-Go Test, s (SD)*

**All participants**

Baseline 717 20.81 (16.77) 16.77 (13.32) 21.87 (14.88)

1.5-year follow-up 529 23.50 (14.60) 19.63 (15.12) 23.75 (15.15)

3-year follow-up 389 25.33 (23.59) 19.13 (14.39) 26.51 (25.78)

5-year follow-up 266 25.44 (17.76) 19.56 (10.12) 19.90 (11.07)

**Men**

Baseline 287 18.11 (13.32) 15.21 (11.79) 17.91 (11.01)

1.5-year follow-up 210 23.82 (16.77) 17.44 (12.95) 19.36 (7.93)

3-year follow-up 149 21.62 (11.12) 16.82 (12.13) 32.97 (46.45)

5-year follow-up 94 18.71 (8.75) 17.65 (9.29) 17.96 (7.80)

**Women**

Baseline 430 22.31 (18.29) 18.03 (14.34) 23.32 (15.89)

1.5-year follow-up 319 23.33 (13.38) 21.37 (16.47) 25.29 (16.77)

3-year follow-up 240 27.12 (27.58) 20.82 (15.66) 24.23 (12.90)

5-year follow-up 172 27.26 (19.19) 20.82 (10.21) 20.67 (12.22)

______________________________________________________________________________________________________________________________

* < 25 nmol/L (severely deficient); ≥ 25 to < 50 nmol/L (deficient); 50 to 74 nmol/L (insufficient), and ≥ 75 nmol/L (sufficient) [5,10], with two middle category collapsed.

**Table S3.** β estimates of grip strength by pre-defined 25(OH)D * categories over 5 years.

| **Outcome** | **Effects/Variable** | **Model 1** |  | **Model 2** |  | **Model 3** |  |
| --- | --- | --- | --- | --- | --- | --- | --- |
|  |  | β (SE) ^†^ | *p* | β (SE) ^†^ | *p* Value | β (SE) ^†^ | *p* |
| *All participants* |  |  |  |  |  |  |  |
| GS (kg) | Intercept | 18.91 (0.35) | < 0.001 | 18.23 (0.75) | < 0.001 | 9.91 (0.87) | < 0.001 |
|  | Pre-defined 25(OH)D category |  |  |  |  |  |  |
|  | Lowest | −2.50 (0.63) | < 0.001 | −1.46 (0.64) | 0.02 | −0.53 (0.45) | 0.24 |
|  | Middle (ref) | 0 |  | 0 |  | 0 |  |
|  | Highest | −3.40 (0.78) | < 0.001 | −3.35 (0.86) | < 0.001 | −0.19 (0.54) | 0.72 |
| GS decline ^‡^ | Time | −0.79 (0.04) | < 0.001 | −0.65 (0.13) | < 0.001 | −0.46 (0.13) | 0.001 |
|  | Time^2^ |  |  | −0.03 (0.03) | 0.27 | −0.03 (0.03) | 0.3 |
| Rate of decline | Slope ^§^ |  |  |  |  |  |  |
|  | 25(OH)D category ×Time |  |  |  |  |  |  |
|  | Lowest × Time |  |  | 0.40 (0.25) | 0.11 | 0.25 (0.25) | 0.32 |
|  | Middle × Time (ref) |  |  | 0 |  | 0 |  |
|  | Highest × Time |  |  | −0.04 (0.31) | 0.89 | −0.21 (0.32) | 0.51 |
|  | 25(OH)D category × Time^2^ |  |  |  |  |  |  |
|  | Lowest × Time^2^ |  |  | −0.11 (0.05) | 0.03 | −0.10 (0.05) | 0.06 |
|  | Middle × Time^2^ |  |  | 0 |  | 0 |  |
|  | Highest × Time^2^ |  |  | −0.001 (0.06) | 0.99 | −0.005 (0.06) | 0.94 |
| *Men* |  |  |  |  |  |  |  |
| GS (kg) | Intercept | 25.30 (0.47) | < 0.001 | 25.13 (1.04) | < 0.001 | 18.50 (1.71) | < 0.001 |
|  | Pre-defined 25(OH)D category |  |  |  |  |  |  |
|  | Lowest | −2.09 (0.93) | 0.03 | −2.39 (0.96) | 0.02 | −0.36 (0.92) | 0.70 |
|  | Middle (ref) | 0 |  | 0 |  | 0 |  |
|  | Highest | −2.28 (1.33) | 0.09 | −2.16 (1.38) | 0.12 | −0.77 (1.27) | 0.55 |
| GS decline ^‡^ | Time | −1.10 (0.08) | < 0.001 | −1.07 (0.22) | < 0.001 | −1.17 (0.22) | < 0.001 |
|  | Time^2^ |  |  | 0.01 (0.04) | 0.86 | 0.02 (0.04) | 0.57 |
| Rate of decline | Slope ^§^ |  |  |  |  |  |  |
|  | 25(OH)D category × Time |  |  |  |  |  |  |
|  | Lowest × Time |  |  | 1.55 (0.47) | 0.001 | 1.23 (0.48) | 0.01 |
|  | Middle × Time (ref) |  |  | 0 |  | 0 |  |
|  | Highest × Time |  |  | −0.19 (0.68) | 0.78 | −0.46 (0.70) | 0.51 |
|  | 25(OH)D category × Time^2^ |  |  |  |  |  |  |
|  | Lowest × Time^2^ |  |  | −0.46 (0.10) | < 0.001 | −0.42 (0.10) | < 0.001 |
|  | Middle × Time^2^ |  |  | 0 |  | 0 |  |
|  | Highest × Time^2^ |  |  | -0.004 (0.14) | 0.98 | 0.04 (0.14) | 0.79 |
| *Women* |  |  |  |  |  |  |  |
| GS (kg) | Intercept | 13.83 (0.27) | < 0.001 | 12.73 (0.56) | < 0.001 | 11.47 (0.86) | < 0.001 |
|  | Pre-defined 25(OH)D category |  |  |  |  |  |  |
|  | Lowest | −1.14 (0.46) | 0.01 | −1.09 (0.50) | 0.03 | −0.59 (0.47) | 0.21 |
|  | Middle (ref) | 0 |  | 0 |  | 0 |  |
|  | Highest | −1.04 (0.53) | 0.05 | −1.11 (0.57) | 0.053 | −0.33 (0.52) | 0.53 |
| GS decline ^‡^ | Time | −0.59 (0.05) | < 0.001 | −0.25 (0.15) | 0.1 | −0.32 (0.15) | 0.04 |
|  | Time^2^ |  |  | −0.07 (0.03) | 0.02 | −0.06 (0.03) | 0.04 |
| Rate of decline | Slope ^§^ |  |  |  |  |  |  |
|  | 25(OH)D category × Time |  |  |  |  |  |  |
|  | Lowest × Time |  |  | -0.21 (0.28) | 0.44 | −0.20 (0.28) | 0.47 |
|  | Middle × Time |  |  | 0 |  | 0 |  |
|  | Highest × Time |  |  | 0.24 (0.33) | 0.46 | −0.24 (0.33) | 0.48 |
|  | 25(OH)D category x Time^2^ |  |  |  |  |  |  |
|  | Lowest × Time^2^ |  |  | 0.05 (0.06) | 0.43 | 0.04 (0.06) | 0.46 |
|  | Middle × Time^2^ |  |  | 0 |  | 0 |  |
|  | Highest × Time^2^ |  |  | 0.03 (0.07) | 0.66 | 0.02 (0.07) | 0.73 |
| *Restricted cohort* |  |  |  |  |  |  |  |
| GS (kg) | Intercept | 19.61 (0.39) | < 0.001 | 19.28 (0.84) | < 0.001 | 9.81 (0.99) | < 0.001 |
|  | Pre-defined 25(OH)D category |  |  |  |  |  |  |
|  | Lowest | −3.12 (0.66) | < 0.001 | −3.20 (0.72) | < 0.001 | −0.56 (0.48) | 0.25 |
|  | Middle (ref) | 0 |  | 0 |  | 0 |  |
|  | Highest | −0.69 (1.22) | 0.57 | −0.58 (0.93) | 0.66 | 0.36 (0.84) | 0.67 |
| GS decline ^‡^ | Time |  |  | −0.74 (0.14) | < 0.001 | −0.51 (0.15) | < 0.001 |
|  | Time^2^ |  |  | −0.02 (0.03) | 0.49 | −0.02 (0.03) | 0.56 |
| Rate of decline | Slope ^§^ |  |  |  |  |  |  |
|  | 25(OH)D category × Time |  |  |  |  |  |  |
|  | Lowest × Time |  |  | 0.48 (0.26) | 0.06 | 0.32 (0.26) | 0.22 |
|  | Middle × Time (ref) |  |  | 0 |  | 0 |  |
|  | Highest × Time |  |  | 0.06 (0.46) | 0.9 | 0.03 (0.47) | 0.95 |
|  | 25(OH)D category × Time^2^ |  |  |  |  |  |  |
|  | Lowest × Time^2^ |  |  | −0.12 (0.05) | 0.03 | −0.11 (0.05) | 0.04 |
|  | Middle × Time^2^ |  |  | 0 |  | 0 |  |
|  | Highest × Time^2^ |  |  | 0.03 (0.09) | 0.76 | −0.03 (0.09) | 0.76 |

* < 25 nmol/L (severely deficient); ≥ 25 to < 50 nmol/L (deficient); 50 to 74 nmol/L (insufficient), and ≥ 75 nmol/L (sufficient) [5,10], with two middle category collapsed. ^†^ β-coefficients (SE) are estimates of fixed effects with longitudinal GS data to evaluate population averages in GS. Fixed effects of covariates estimated initial level and trajectory differences in GS as a function of the covariate in the model. ^‡^ The main effect of time (Time and Time^2^) tested linear and non-linear (quadratic) change in GS over 5 years. ^§^ Interaction terms tested whether GS slopes varied by the covariate over 5 years. Model 1 includes a linear trend of time and pre-defined 25(OH)D categories. Model 2 is additionally adjusted for quadratic trend of time, interaction terms (Time × 25(OH)D categories, Time^2^ × 25(OH)D categories) and season of blood draw. Model 3 is further adjusted for sex, anthropometry (height and FFM), health-related variables (cognitive impairment, disease count, and self-rated health), physical activity, and interaction term (sex × Time).

**Table S4.** β estimates of Timed Up-and-Go test by pre-defined 25(OH)D * categories over 5 years.

| **Outcome** | **Effects/Variable** | **Model 1** |  | **Model 2** |  | **Model 3** |  |
| --- | --- | --- | --- | --- | --- | --- | --- |
|  |  | β (SE) ^†^ | *p* | β (SE) ^†^ | *p* | β (SE) ^†^ | *p* |
| *Entire cohort* |  |  |  |  |  |  |  |
| TUG (log_10_ s) | Intercept | 1.17 (0.01) | < 0.001 | 1.17 (0.02) | < 0.001 | 1.54 (0.03) | < 0.001 |
|  | Pre-defined 25(OH)D category |  |  |  |  |  |  |
|  | Lowest | 0.10 (0.02) | < 0.001 | 0.09 (0.02) | < 0.001 | 0.02 (0.02) | 0.25 |
|  | Middle (ref) | 0 |  | 0 |  | 0 |  |
|  | Highest | 0.10 (0.02) | < 0.001 | 0.11 (0.03) | < 0.001 | 0.04 (0.02) | 0.053 |
| TUG decline ^‡^ | Time | 0.03 (0.002) | < 0.001 | 0.06 (0.06) | < 0.001 | 0.06 (0.006) | < 0.001 |
|  | Time^2^ |  |  | −0.005 (0.001) | < 0.001 | −0.007 (0.001) | < 0.001 |
| Rate of decline | Slope ^§^ |  |  |  |  |  |  |
|  | 25(OH)D category × Time |  |  |  |  |  |  |
|  | Lowest × Time |  |  | 0.02 (0.01) | 0.08 | 0.02 (0.01) | 0.03 |
|  | Middle × Time (ref) |  |  | 0 |  | 0 |  |
|  | Highest × Time |  |  | 0.006 (0.01) | 0.7 | 0.01 (0.01) | 0.34 |
|  | 25(OH)D category x Time^2^ |  |  |  |  |  |  |
|  | Lowest × Time^2^ |  |  | −0.003 (0.002) | 0.15 | −0.004 (0.002) | 0.08 |
|  | Middle × Time^2^ |  |  | 0 |  | 0 |  |
|  | Highest × Time^2^ |  |  | −0.003 (0.003) | 0.4 | −0.004 (0.003) | 0.2 |
| *Men* |  |  |  |  |  |  |  |
| TUG (log_10_ s) | Intercept | 1.13 (0.02) | < 0.001 | 1.13 (0.03) | < 0.001 | 1.56 (0.05) | < 0.001 |
|  | Pre-defined 25(OH)D category |  |  |  |  |  |  |
|  | Lowest | 0.1 (0.03) | 0.001 | 0.08 (0.03) | 0.01 | −0.01 (0.03) | 0.65 |
|  | Middle (ref) | 0 |  | 0 |  | 0 |  |
|  | Highest | 0.08 (0.04) | 0.05 | 0.07 (0.04) | 0.13 | −0.02 (0.04) | 0.68 |
| TUG decline^‡^ | Time | 0.05 (0.003) |  | 0.05 (0.01) | < 0.001 | 0.05 (0.008) | < 0.001 |
|  | Time^2^ |  |  | −0.004 (0.002) | 0.01 | −0.006 (0.002) | < 0.001 |
| Rate of decline | Slope ^§^ |  |  |  |  |  |  |
|  | 25(OH)D category × Time |  |  |  |  |  |  |
|  | Lowest × Time |  |  | 0.06 (0.02) | 0.003 | 0.07 (0.02) | < 0.001 |
|  | Middle × Time (ref) |  |  | 0 |  | 0 |  |
|  | Highest × Time |  |  | 0.05 (0.02) | 0.06 | 0.05 (0.02) | 0.03 |
|  | 25(OH)D category × Time^2^ |  |  |  |  |  |  |
|  | Lowest × Time^2^ |  |  | −0.004 (0.001) | 0.01 | −0.01 (0.002) | < 0.001 |
|  | Middle × Time^2^ |  |  | 0 |  | 0 |  |
|  | Highest × Time^2^ |  |  | −0.01 (0.005) | 0.07 | −0.01 (0.005) | 0.04 |
| *Women* |  |  |  |  |  |  |  |
| TUG (log_10_ s) | Intercept | 1.20 (0.01) | < 0.001 | 1.21 (0.03) | < 0.001 | 1.50 (0.04) | < 0.001 |
|  | Pre-defined 25(OH)D category |  |  |  |  |  |  |
|  | Lowest | 0.08 (0.03) | 0.001 | 0.08 (0.03 ) | 0.004 | 0.04 (0.02) | 0.08 |
|  | Middle (ref) | 0 |  | 0 |  | 0 |  |
|  | Highest | 0.09 (0.03) | 0.003 | 0.1 (0.03) | 0.001 | 0.06 (0.02) | 0.02 |
| TUG decline^‡^ | Time | 0.03 (0.003) | < 0.001 | 0.06 (0.008) | < 0.001 | 0.06 (0.008) | < 0.001 |
|  | Time^2^ |  |  | −0.006 (0.001) | < 0.001 | −0.008 (0.002) | < 0.001 |
| Rate of decline | Slope ^§^ |  |  |  |  |  |  |
|  | 25(OH)D category × Time |  |  |  |  |  |  |
|  | Lowest × Time |  |  | 0.08 (0.03) | 0.004 | −0.00003 (0.002) | 1.0 |
|  | Middle × Time |  |  | 0 |  | 0 |  |
|  | Highest × Time |  |  | 0.1 (0.03) | 0.001 | −0.003 (0.02) | 0.87 |
|  | 25(OH)D category × Time^2^ |  |  |  |  |  |  |
|  | Lowest × Time^2^ |  |  | −0.0003 (0.003) | 0.92 | −0.0002 (0.003) | 0.95 |
|  | Middle × Time^2^ |  |  | 0 |  | 0 |  |
|  | Highest × Time^2^ |  |  | 0.0002 (0.004) | 0.96 | −0.001 (0.004) | 0.74 |
| *Restricted cohort* |  |  |  |  |  |  |  |
| TUG (log_10_ s) | Intercept | 1.16 (0.01) | < 0.001 | 1.15 (0.02) | < 0.001 | 1.54 (0.0.03) | < 0.001 |
|  | Pre-defined 25(OH)D category |  |  |  |  |  |  |
|  | Lowest | 0.11 (0.02) | < 0.001 | 0.1 (0.02) | < 0.001 | 0.02 (0.02) | 0.13 |
|  | Middle (ref) | 0 |  | 0 |  | 0 |  |
|  | Highest | 0.001 (0.03) | 0.98 | −0.005 (0.04) | 0.89 | −0.01 (0.03) | 0.61 |
| TUG decline ^‡^ | Time | 0.03 (0.002) | < 0.001 | 0.06 (0.006) | < 0.001 | 0.06 (0.006) | < 0.001 |
|  | Time^2^ |  |  | −0.005 (0.001) | < 0.001 | −0.007 (0.001) | < 0.001 |
| Rate of decline | Slope ^§^ |  |  |  |  |  |  |
|  | 25(OH)D category × Time |  |  |  |  |  |  |
|  | Lowest × Time |  |  | 0.02 (0.01) | 0.09 | 0.02 (0.01) | 0.05 |
|  | Middle × Time (ref) |  |  | 0 |  | 0 |  |
|  | Highest × Time |  |  | 0.02 (0.02) | 0.38 | 0.02 (0.02) | 0.42 |
|  | 25(OH)D category × Time^2^ |  |  |  |  |  |  |
|  | Lowest × Time^2^ |  |  | −0.003 (0.002) | 0.15 | −0.004 (0.002) | 0.08 |
|  | Middle × Time^2^ |  |  | 0 |  | 0 |  |
|  | Highest × Time^2^ |  |  | −0.005 (0.004) | 0.23 | −0.005 (0.004) | 0.23 |

* < 25 nmol/L (severely deficient); ≥ 25 to < 50 nmol/L (deficient); 50 to 74 nmol/L (insufficient), and ≥ 75 nmol/L (sufficient) [5,10], with two middle category collapsed. ^†^ β-coefficients (SE) are estimates of fixed effects with longitudinal log_10_ transformed TUG data to evaluate population averages in TUG time. Fixed effects of covariates estimated initial level and trajectory differences in TUG as a function of the covariate in the model. ^‡^ The main effect of time (Time and Time^2^) tested linear and non-linear (quadratic) change in TUG over 5 years. ^§^ Interaction terms tested whether TUG slopes varied by the covariate over 5 years. Model 1 includes a linear trend of time and season-specific 25(OH)D categories. Model 2 is additionally adjusted for quadratic trend of time and interaction terms (Time × 25(OH)D categories, Time^2^ × 25(OH)D categories). Model 3 is further adjusted for sex, anthropometry (height and FFM), health-related variables (cognitive impairment, disease count, self-rated health), physical activity, use of walking aids during TUG testing (time-varying covariate) and interaction term (sex × Time).
